# Gap Reconstruction in Optical Motion Capture Sequences Using Neural Networks

**DOI:** 10.3390/s21186115

**Published:** 2021-09-12

**Authors:** Przemysław Skurowski, Magdalena Pawlyta

**Affiliations:** 1 Department of Graphics, Computer Vision and Digital Systems, Faculty of Automatic Control, Electronics and Computer Science, Silesian University of Technology, Akademicka 16, 44-100 Gliwice, Poland; Magdalena.Pawlyta@polsl.pl; 2 Polish-Japanese Academy of Information Technology, Koszykowa 86, 02-008 Warsaw, Poland

**Keywords:** motion capture, neural networks, reconstruction, gap filling, FFNN, LSTM, BILSTM, GRU

## Abstract

Optical motion capture is a mature contemporary technique for the acquisition of motion data; alas, it is non-error-free. Due to technical limitations and occlusions of markers, gaps might occur in such recordings. The article reviews various neural network architectures applied to the gap-filling problem in motion capture sequences within the FBM framework providing a representation of body kinematic structure. The results are compared with interpolation and matrix completion methods. We found out that, for longer sequences, simple linear feedforward neural networks can outperform the other, sophisticated architectures, but these outcomes might be affected by the small amount of data availabe for training. We were also able to identify that the acceleration and monotonicity of input sequence are the parameters that have a notable impact on the obtained results.

## 1. Introduction

Motion capture (mocap) [1,2], in recent years, has become a mature technology that has an important role in many application areas. Its main application is in computer graphics, where it is applied in gaming and movie FX for the generation of realistic-looking character animation. Other prominent applications areas are biomechanics [3], sports [4], medical sciences (involving biomechanical [5] and the other branches, i.e., neurology [6]), and rehabilitation [7].

Optical motion capture (OMC) relies on the visual tracking and triangulation of active or retro-reflective passive markers. Assuming a rigid body model, successive positions of markers (trajectories) are used in further stages of processing to drive an associated skeleton, which is used as a key model for the animation of human-like or animal characters.

OMC is commonly considered the most reliable mocap technology; it is sometimes called the ‘gold standard’, as it outperforms the other mocap technologies. However, the process of acquiring marker locations is not error-free. Noise, which is immanent in any measurement system, has been studied in numerous works [8,9], which suggests it is not just simple additive Gaussian process. The noise types present in OMC systems were identified in [10]; these are red, pink, white, blue-violet, and Markov–Gaussian-correlated noises; however, they are not a big issue for the mocap operators since they have rather low amplitudes and can be quite efficiently filtered out. The most annoying errors come from marker observation issues. They occur due to marker occlusion and the marker leaving the scene, and result in a lack of the recorded data-gaps that are typically represented as not a number (NaN) values.

The presence of gaps is common and results in everyday praxis, which requires painstaking visual trajectory examination and manual trajectory editing by operators. This can be assisted by software support for trajectory reconstruction.

In this work, we propose a marker-wise approach that addresses the trajectory reconstruction problem. We analyze the usability of various neural network architectures applied to regressive tasks. The regression/prediction exploits inter-marker correlations between markers placed on the same body parts. Therefore, we employed a functional body mesh structure (FBM) [11] as a framework to model the kinematic structure of the subject. I Thisan be calculated ad-hoc for any articulated subject or rigid objects, so we do not need a skeleton model.

The article is organized as follows: in Section 2, we disclose the background for the article—mocap pipeline with sources of distortion and former works on the distortions in optical mocap systems; Section 3 describes the proposed method, with its rationales and design considerations, and experiment plan. In the Section 4 we provide results, and a discussion and interpretation of results. Section 5 summarizes the article.

## 2. Background

### 2.1. Optical Motion Capture Pipeline

**Figure 1:**
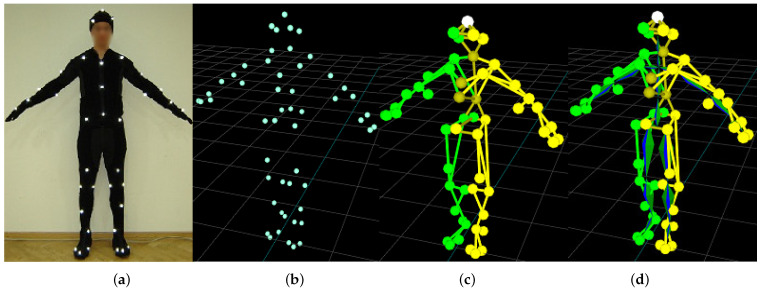
Stages of the motion capture pipeline: actor (**a**); registered markers (**b**); body mesh (**c**); mesh matched skeleton (**d**).

Optical motion capture systems track the markers—usually passive retro-reflective spheres in near-infrared images (NIR) images. The basic pipeline is shown in Figure 1. The markers are observed by several geometrically calibrated NIR cameras. The visual wavelengths cut-off, and, hence, the images, contain just white dots, which are matched between the views and triangulated, so the outcome of the early stage of mocap is a time series containing Cartesian coordinates of all markers. An actor and/or object wears a sufficient number of markers to represent body segments—marker layout usually follows a predefined layout standard. The body segments are represented by a predefined mesh, which identifies the body segments and is a marker-wise representation of body structure. Finally, mocap recording takes the form of a skeleton angle time series, which represents the mocap sequence as orientations (angles) in joints and a single Cartesian coordinate for body root (pelvis usually).

### 2.2. Functional Body Mesh

Functional body mesh (FBM) is a authors’ original contribution, that forms a framework for marker-wise mocap data processing, which incorporates also the kinematic structure of a represented object. The FBM structure is not given in advance, but it can be inferred based on the articulated object representative motions [11]. For human actors it resembles standard meshes, but it can be applied for virtually any vertebrates. It assumes the body is divided into rigid segments (submeshes), which are organized into a tree structure. The model represents the hierarchy of subjects’ kinematic structure, reflecting bonds between body segments, where every segment is a local rigid body model—usually based on an underlying bone.

**Figure 2:**
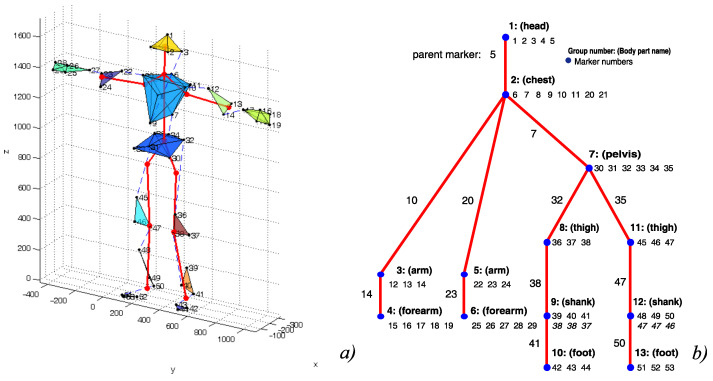
Outline of the body model (**a**), and corresponding parts hierarchy annotated with parents and siblings (**b**).

The rigid segments maintain the distance between the markers and, additionally, for each child segment, one representative marker is assumed within the parent one, which is also assumed to maintain a constant distance from the child markers. The typical FBM for the human actor is shown in Figure 2b as a tree. The segments and constituent markers are located in nodes, whereas the parent marker is denoted on the parent–child edge.

### 2.3. Previous Works

Gap filling is a classical problem frequently addressed in research on mocap technologies. It was in numerous works, which proposed various approaches. The existing methods can be divided into three main groups—skeleton-based, marker-wise, and coordinate-based.

A classical skeleton-based method was proposed by Herda et al. [12], they estimate skeleton motion and regenerate markers on the body envelope. Aristidou and Lanesby [13] proposed the other method based on a similar concept, where the skeleton is a source for constraints in inverse kinematics estimation of marker location. Also, Perepichka et al. [14] combined IK of skeleton model with deep NN to detect erroneously located markers and to place them on a probable trajectory. All aforementioned approaches require either to have a predefined skeleton or to infer the skeleton as the entry step of an algorithm.

The skeleton-free methods consider information from markers only, usually acknowledging the whole sequence as a single multivariable (matrix), thus losing the kinematic structure of the represented actor. They rely on various concepts, starting from the simple interpolating methods [15,16,17]. The proposal by Liu and McMillan [18] employed ‘local’ (neighboring markers) low-dimensional least squares models combined with PCA for missing marker reconstruction. A significant group of gap reconstruction proposals is based on the low-rank matrix completion methods. They employ various mathematical tools (e.g., matrix factorization with SVD) for the missing data completion, relying on inter marker correlations. Among the others, these methods are described in the following works [19,20]. Another approach is somewhat related: it is a fusion of several regressions and interpolation methods, which was proposed in [21].

Predicting markers (or joint) position is another concept that is the basis of gap-filling techniques. One such concept is a predictive model by Piazza et al. [22], which decomposes the motion into linear and circular and finds momentary predictors by curve fitting. More sophisticated dynamical models based on the Kalman filter (KF) are commonly applied. Wy and Boulanger [23] proposed a KF with velocity constraints; however, this achieved moderate success due to drift. A KF with an expectation-maximization algorithm was also used in two related approaches by Li et al.—DynaMMo [24], and BoLeRO [25] (the latter is actually Dynammo with bone length constraints). Another approach was proposed by Burke and Lanesby [26], who applied dimensionality reduction by PCA and then Kalman smoothing for the reconstruction of missing markers.

Another group of methods is dictionary-based. These algorithms recover the trajectories using a dictionary created from previously recorded sequences. They result in satisfactory outcomes as long the specific motion is in the database. They are represented by the works of Wang et al. [27], Aristidou et al. [28], and Zhang and van de Panne [29].

Finally, neural networks are another group of methods used in marker trajectory reconstruction. The task can be described as a sequence-to-sequence regression problem, whereas NN applied for regression has been recognized since the early 1990s in the work of Hornik [30]; hence, NN seems to be a natural choice for the task. Surprisingly, however, they become popular quite late. In the work of Fragkiadaki et al. [31], an encoder–recurrent-decoder (ERD) was proposed, employing long-short term memory (LSTM) as a recurrent layer. A similar approach (ERD) was proposed by Harvey et al. [32] for in-between motion generation on the basis of asmall amount of keyframes. Mall et al. [33] modified the ERD and proposed an encoder–bidirectional-filter (EBF) based on the bidirectional LSTM (BILSTM). In the work of Kucharenko et al. [34], a classical two-layer LSTM and window-based feed-forward NN (FFNN) were employed. A variant of ResNet is applied by Holden [35] to reconstruct marker positions from noisy data as a ttrajectory reconstruction task. A set of extensions to the plain LSTM were proposed by Ji et al. [36]; they introduced attention (a weighting mechanism) and LS-derived spatial constraints, which result in an improvement in performance. Convolution auto-encoders was proposed by Kaufmann et al. [37].

## 3. Materials and Methods

### 3.1. Proposed Regression Approach

The proposed approach involves employing various neural networks architectures for the regression task. These are FFNN and three variants of contemporary recursive neural networks—gated recurrent unit (GRU), long-short-term memory (LSTM), and bidirectional LSTM (BILSTM). In our proposal, these methods predict trajectories of lost markers on the basis of a local dataset—the trajectories of neighboring markers.

The proposed utilization procedure of NN differs from the scenario that is typically employed in machine learning. We do not feed the NNs with a massive amount of training sequences in advance to form a predictive model. Instead, we consider each sequence separately and try to reconstruct the gaps in individual motion trajectory on the basis of its own data only. This makes sense as long as the marker motion is correlated and most of the sequence is correct and representative enough. This is the same as for the other common regression methods, starting with the least squares. Therefore, the testing data are the whole ‘lost’ segment (gap), whereas the training is the remaining part of the trajectory. Depending on the gap sizes, and sequence length used in the experiment, the testing can be between 0.6% (for short gaps and long sequences) and up to 57.1% (for long gaps in short sequences).

The selection of such a non-typical approach requires a justification. It is likely that training the NN models for prediction of marker position in a conventional way, using a massive dataset of mocap sequences, would be able to generalize enough to adjust to different body sizes and motions. However, it will be tightly coupled with the marker configuration, not to mention the other actors, such as animals. The other issue is obtaining such a large amount of data. Despite our direct access to the lab resources, this is still quite a cumbersome task, since we believe these might be not enough, especially as the resources available online from various other labs are hardly usable, since they employ different marker setups.

The forecasting of timeseries is a typical problem addressed by RNNs [38]. Usually, numerous training and testing sequences allow for a prediction of the future states of the modelled system (e.g., power consumption or remaining useful life of devices). A more similar situation, where RNNs are also applied, is forecasting the time series for problems lacking massive training data (e.g., COVID-19 [39]). An analysis of LSTM architectures for similar cases is presented in [40]. However, in these works, the forecast of future values is based on the past values. What makes our case a bit different is the fact that we usually have to predict the value in-the-middle, so the past and future values are available.

#### 3.1.1. Feed Forward Neural Network

**Figure 3:**
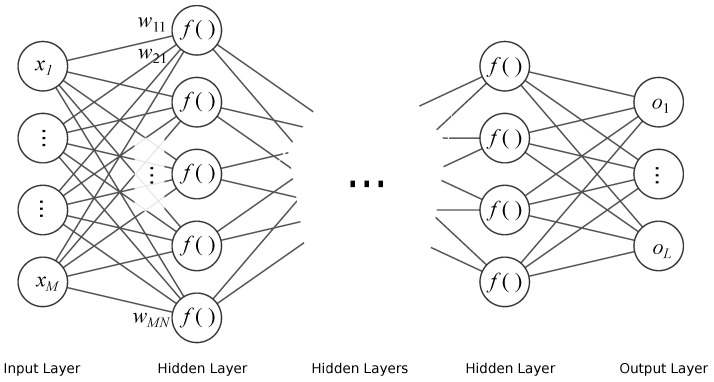
Schematic of FFNN.

FFNN is the simplest neural network architecture. In this architecture, the information flows in one direction, as its structure forms an acyclic directed graph. The neurons are modeled in the nodes with activation functions (usually sigmoid) using the weighted sum of inputs. These networks are typically organized into layers, where the output from the previous layer becomes an input to a successive one. This architecture of networks is employed for regression and classification tasks, either alone or as final stages in a larger structures (such as modern deep NN). The architecture of the NN that we employed is shown in Figure 3. The basic equation (output) of a single—*k*-th artificial neuron is given as:

(1) $$y_{k}\left(x\right)=f\left(\sum_{j}w_{jk}x_{j}+b\right),$$

where $x_{j}$ is *j*-th input, $w_{kj}$ is *j*-th input weight, *b*—a bias value, *f*—is transfer (activation) function. Transfer function depends on the layer purpose; these are typically a sigmoid for hidden layers, threshold, linear, or softmax for final layers (for regression and classification problems, respectively), or others.

#### 3.1.2. Recurrent Neural Networks

**Figure 4:**
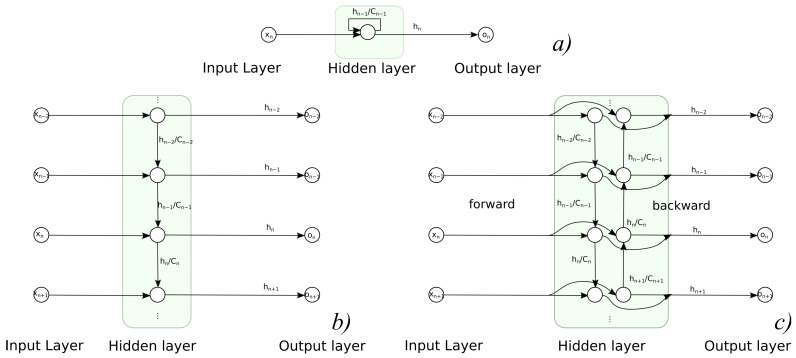
Usage of recurrent NNs in sequence to sequence task: (**a**) folded, (**b**) unfolded unidirectional variant, (**c**) unfolded bidirectional variant.

Recurrent neural networks (RNN) are the types of architecture that employ cycles in NN structure; this allows for the consideration of current input value as well as preserving the previous inputs and internal states of NN in memory (and future ones for bidirectional architecture). Such an approach allows for NN to deal with timed processes and to recognize process dynamics, not just static values—it applies to such tasks as a signal prediction or recognition of sequences. Regarding the applicability, aside from classic problem dichotomy (classification and regression), RNN results might need another task differentiation. One must decide whether the task is a sequence-to-one or sequence-to-sequence problem, so the network has to return either a single result for the whole sequence or a single result for each data tuple in sequence. The prediction/regression task is a sequence-to-sequence problem, as demonstrated with RNNs in Figure 4 in different variants—both folded and unfolded, uni- and bi-directional.

**Figure 5:**
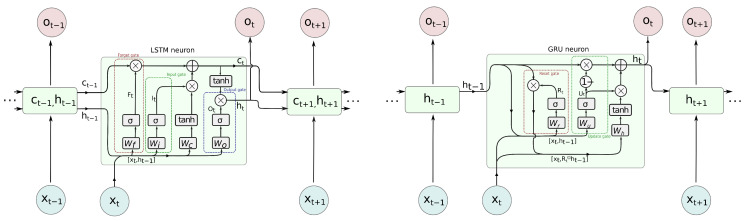
LSTM (**left**) and GRU (**right**) neurons in detail.

At present two types of neuron are predominantly applied in RNN–long short term memory (LSTM) and gated recurrent unit (GRU), of which the former is also applied in bidirectional variant (BILSTM). They evolved from a plain RNN called ‘vanilla’, and they prevent vanishing gradient problems when back-propagating errors in the learning process. Their detailed designs are unfolded in Figure 5. These cell types rely on the input information and information from previous time steps, and those previous states are represented in various ways. GRU passes an output (hidden signal *h*) between the steps, whereas LSTM also passes a *h* and internal cell state *C*. These values are interpreted as memory—*h* as short term, and *C* as long term. Their activation function is typical sigmoid, which is modeled with a hyperbolic tangent (tanh), but there are additional elements present in the cell. The contributing components, such as input or previous values, are subject to *‘gating’*—their share is controlled by Hadamard product (element-wise product denoted as ⊙ or ⊗ in diagram) with 0–1 sigmoid function $\sigma \left(x\right)=\frac{1}{1+e^{-x}}$. The individual σ values are obtained by weighted input and state values.

In more detail, in LSTM, we pass two variables h,C and have three gates—forget, input and output. They govern how much of the respective contribution passes to further processing. The forget gate ($f_{t}$) decides how much of the past cell internal state ($C_{t-1}$) is to be kept; the input gate ($i_{t}$) controls how much new contribution ${\tilde{C}}_{t}$ caused by input ($x_{t}$) annd taken into the current cell state ($C_{t}$). Finally, the output gate ($o_{t}$) controls what part of activation is based on the cell internal state; ($C_{t}$) is taken as cell output ($h_{t}$). The equations are as follows:

(2) $$\begin{array}{cc} f_{t} & =\sigma (W_{f}\cdot \left[x_{t},h_{t-1}\right]+b_{f}), \end{array}$$

(3) $$\begin{array}{cc} i_{t} & =\sigma (W_{i}\cdot \left[x_{t},h_{t-1}\right]+b_{f}), \end{array}$$

(4) $$\begin{array}{cc} {\tilde{C}}_{t} & =\tanh(W_{c}\cdot \left[x_{t},h_{t-1}\right]+b_{c}), \end{array}$$

(5) $$\begin{array}{cc} C_{t} & =f_{t}⊙C_{t-1}+i_{t}⊙{\tilde{C}}_{t}, \end{array}$$

(6) $$\begin{array}{cc} o_{t} & =\sigma (W_{o}\cdot \left[x_{t},h_{t-1}\right]+b_{f}), \end{array}$$

(7) $$\begin{array}{cc} h_{t} & =o_{t}⊙\tanh\left(C_{t}\right). \end{array}$$

The detailed schematic of GRU is a bit simpler. Only one signal, hidden (layer output) value (*h* for *hi*), is passed between steps. There are two gates present—the reset gate ($r_{t}$), which controls how much past output ($h_{t-1}$) contributes to the overall cell activation, and the update gate ($u_{t}$), which controls how much current activation (${\tilde{h}}_{t}$) contributes to the final cell output.The above are described by the following equations:

(8) $$\begin{array}{cc} u_{t} & =\sigma (W_{u}\cdot \left[x_{t},h_{t-1}\right]+b_{u}), \end{array}$$

(9) $$\begin{array}{cc} r_{t} & =\sigma (W_{u}\cdot \left[x_{t},h_{t-1}\right]+b_{u}), \end{array}$$

(10) $$\begin{array}{cc} {\tilde{h}}_{t} & =\tanh\left(W_{h}\cdot \left[x_{t},r_{t}⊙h_{t-1}\right]+b_{h}\right), \end{array}$$

(11) $$\begin{array}{cc} h_{t} & =\left(1-u_{t}\right)⊙h_{t-1}+u_{t}⊙{\tilde{h}}_{t}. \end{array}$$

#### 3.1.3. Employed Reconstruction Methods

**Figure 6:**
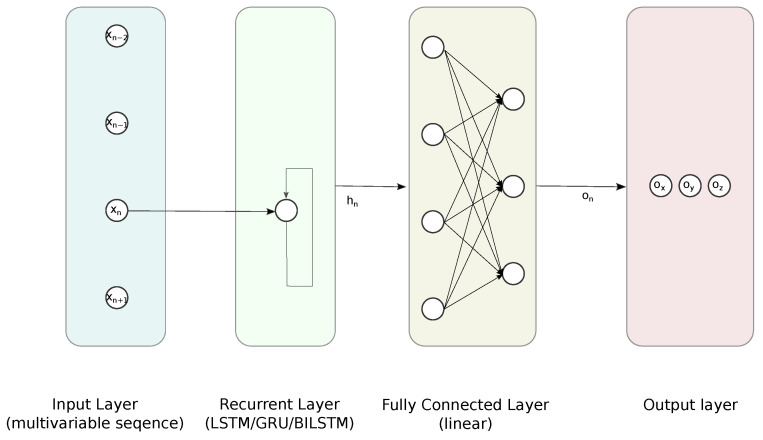
Proposed RNN-FC architecture for the regression task.

We compared the performance of five architectures of NN—two variants of FFNN and three RNN-FCs based on GRU, LSTM, and BILSTM; the outline of the latter is depicted in Figure 6. The detailed structures and hyperparameters of NNs were established empirically, since there are no strict rules or guidelines. Usually, this requires simulating, with parameters sweeping the domain of feasible numbers of layers and neurons [41]. We shared this approach and reviewed the performance of NN using the test data.

- FFNN${}_{\mathrm{lin}}$, with 1 hidden fully connected (FC) layer—containing 8 linear neurons;
- FFNN${}_{\tanh}$, with 1 hidden FC layer—containing 8 sigmoidal neurons;
- LSTM followed by 1 FC layer containing 8 sigmoidal neurons;
- GRU followed by 1 FC layer containing 8 sigmoidal neurons;
- BILSTM followed by 1 FC layer containing 8 sigmoidal neurons.

The output is three valued x,y,z vectors, containing reconstructed marker coordinates.

#### 3.1.4. Implementation Details

The training process was performed using 600 epochs, with the SGDM solver running on the GPU. It involved the whole input sequence with gaps excluded. There was a single instance of sequence in the batch. The sequence parts containing gaps were used as the test data; the remainder was used for training—therefore, the relative size of test part varies between 0.6% and 57.1%. The other parameters are:

- Initial Learn Rate: 0.01;
- Learn Rate Drop Factor: 0.9;
- Learn Rate Drop Period: 10;
- Gradient Threshold 0.7;
- Momentum: 0.8.

We also applied z-score normalization for the input and target data.

Additionally, for comparison, we used a pool of other methods, which should provide nice results for short-term gaps. These are interpolations: linear, spline, modified Akima (makima), piecewise cubic hermite interpolating polynomial (pchip), and the low-rank matrix completion method (mSVD0). All but linear interpolation methods are actually variants of piecewise Hermite cubic polynomial interpolations, which differ in the details of how they compute interpolant slopes. Spline is a generic method, whereas pchip tries to preserve shape, and makima avoids overshooting. However, mSVD [42] is an iterative method decomposing motion capture data with SVD and neglecting the least significant part of the basis transformed signal, reconstructing the original data with replacing missing values using reconstructed ones. The procedure finishes when convergence is reached. We implemented the algorithm, as outlined in [24].

The implementation of methods and experiments was carried out in Matlab 2021a using its implementations of numerical methods and deep learning toolbox.

### 3.2. Input Data Preparation

Constructing the predictor for certain markers, we obtained the locations from all the sibling markers and a single parent one, as they are organized within an FBM structure. For *j*-th marker ($X_{j}=\left[x_{j},y_{j},z_{j}\right]$), we consider parent ($X_{p}$) and sibling markers ($X_{s1},\ldots ,X_{sL}$). To form an input vector, we take two of their values—one for the current moment and with one sample lag. The other variants with more lags or values raised to the higher powers were considered, but after preliminary tests, we neglected them since they did not improve performance.

Each input vector *T*, for the moment *n*, is quite long and is assembled of certain parts, as given below:

(12) $$\begin{array}{cc} \; & \;T\left(n,*\right)=\left[\begin{array}{c} \;\overset{\mathrm{current}\mathrm{and}\mathrm{former}\mathrm{values}\mathrm{of}\mathrm{parent}\mathrm{marker}\;(p)}{\overbrace{x_{p}\left(n\right),y_{p}\left(n\right),z_{p}\left(n\right),x_{p}\left(n-1\right),y_{p}\left(n-1\right),z_{p}\left(n-1\right)}}\;,\;\;\;\;\\ \;\;\;\;\overset{\mathrm{current}\mathrm{and}\mathrm{former}\mathrm{value}\mathrm{of}\mathrm{first}\mathrm{sibling}s_{1}}{\overbrace{x_{s1}\left(n\right),y_{s1}\left(n\right),z_{s1}\left(n\right),x_{s1}\left(n-1\right),y_{s1}\left(n-1\right),z_{s1}\left(n-1\right)}},\\ \vdots \\ \;\;\;\;\;\;\underset{\mathrm{current}\mathrm{and}\mathrm{former}\mathrm{value}\mathrm{of}\mathrm{last}\mathrm{sibling}s_{L}}{\underbrace{x_{sL}\left(n\right),y_{sL}\left(n\right),z_{sL}\left(n\right),x_{sL}\left(n-1\right),y_{sL}\left(n-1\right),z_{sL}\left(n-1\right)}} \end{array}\right]. \end{array}$$

Finally, the input and output data are z-score standardized—zero centered and standard deviation scaled to 1, since such a step notably improves the final results.

### 3.3. Test Dataset

For testing purposes, we used a dataset (Table 1) acquired for professional purposes in the motion-capture laboratory. The ground truth sequences were obtained at the PJAIT human motion laboratory using the industrial-grade Vicon MX system. The system capture volume was 9 m × 5 m × 3 m. To minimize the impact of external interference such as infrared interference from sunlight or vibrations, all windows were permanently darkened and cameras were mounted on scaffolding instead of tripods. The system was equipped with 30 NIR cameras manufactured by Vicon: MX-T40, Bonita10, Vantage V5—wth 10 pieces of each kind.

During the recording, we employed a standard animation pipeline, where data were obtained with Vicon Blade software using a 53-marker setup. The trajectories were acquired at 100 Hz and, by default, they were processed in a standard, industrial-quality way, which includes manual data reviewing, cleaning and denoising, so they can be considered distortion-free.

Several parameters for the test sequences are also presented in Table 2. We selected these parameters as one could consider them to potentially describe prediction difficulty. They are various, and based on different concepts such as information theory, statistics, kinematics, and dynamics, but all characterize the variability in the Mocap signal. They are usually the average value per marker, except for standard deviation (std dev), which reports value per coordinate.

Two non-obvious measures are enumerated: monotonicity and complexity. The monotonicity indicates, on average, the extent to which the coordinate is monotonic. For this purpose, we employed an average Spearman rank correlation, which can be described as follows:

(13) $$monotonicity=\frac{1}{M}\sum_{m=1}^{M}\mathrm{corr}\left(\mathrm{rank}\left(X_{i}\right),1\ldots N\right),$$

where $X_{m}$ is *m*th coordinate, *M* is number of coordinates, *N* is sequence length.

Complexity, on the other hand, is how we estimate the variability of poses in the sequence. For that purpose, we employed PCA, which identifies eigenposes as a new basis for the sequence. The corresponding eigenvalues describe how much of the overall variance is described by each of the eigenposes. Therefore, we decided to take the remainder of the fraction of variance described by the sum of the five largest eigenvalues ($\lambda_{i}$) as a term describing how complex (or rather simple) the sequence is—the simpler the sequence, the more variance is described, with a few eigenposes. Therefore, our complexity measure is simply given as:

(14) $$complexity=1-\sum_{i=1}^{5}\lambda_{i}/\sum_{i=1}^{M}\lambda_{i},$$

where *M* is a number of coordinates.

### 3.4. Quality Evaluation

The natural criterion for the reconstruction task is root mean square error (RMSE), which, in our case, is calculated only for the time and marker, where the gaps occur:

(15) $$\mathrm{RMSE}=\sqrt{\frac{1}{\left|W\right|}\sum_{i\in W}{\left({\hat{X}}_{i}-X_{i}\right)}^{2}},$$

where *W* is a gap map, logically indexing locations of gaps, $\hat{X}$ is a reconstructed coordinate, *X* is the original coordinate.

Additionally, we calculated RMSEs for individual gaps. Local RMSE is a variant of the above formula, and simply given as:

(16) $$RMSE_{k}=\sqrt{\frac{1}{|w_{k}|}\sum_{i\in w_{k}}{\left({\hat{X}}_{i}-X_{i}\right)}^{2}},$$

where $w_{k}\subset W$ is a single gap map logically indexing the location of *k*-th gap, $\hat{X}$ is reconstructed coordinate, *X* is original coordinate. $RMSE_{k}$ is intended to reveal variability in reconstruction capabilities; hence, we used it to obtain statistical descriptors—mean, median, mode, and quartiles and interquartile range.

A more complex evaluation of regression models can be based on infromation criteria. These quality measures incorporate squared error and a number of tunable parameters, as they were designed by searching for a tradeoff between the number of tunable parameters and the obtained error. The two most popular ones are Bayesian Information Criterion (BIC) and Akaike Information Criterion (AIC). BIC is calculated as:

(17) BIC=nlog(MSE)+plog(n),

whereas AIC formula is as follows:

(18) AIC=nlog(MSE)+2p,

where: mean squared error $\mathrm{MSE}={\mathrm{RMSE}}^{2}$, *n* is a number of testing data, *p* is a number of tunable parameters.

### 3.5. Experimental Protocol

During the experiments, we simulated gap occurrence in perfectly reconstructed source sequences. We simulated gaps of different average lengths—10, 20, 50, 100 and 200 samples (0.1, 0.2, 0.5, 1, and 2 s, respectively). The assumed gap sizes were chosen to represent situations of various levels of difficulty, from short-and-simple to difficult ones, when gaps are long. For every gap length, we performed 100 simulation iterations, where the training and testing data do not intermix between simulation runs. The steps performed in every iteration are as follows:

1. We introduce two gaps of assumed length (on average) to the random markers at random moments; actual values are stored as testing data;
2. The model is trained using the remaining part of the sequence (all but gaps);
3. We reconstruct (predict) the gaps using the pool of methods;
4. The resulting values are stored for evaluation.

We report the results as RMSE and descriptive statistical descriptors for $RMSE_{k}$ for every considered reconstruction technique. Additionally, we verified the correlation between RMSE and the variability descriptors for sequences. It is intended to reveal what are the sources of difficulties in predicting the marker trajectories.

#### Gap Generation Procedure

The procedure of gap contamination, which was employed, introduces distortions into the sequences in a controlled way. The parameter characterizing the experiment is an average-length number of occurrences of gaps. the sequence of operations distorting the signal is as follows: at first, we draw moments to contaminate, then select a random marker. The duration of distortions and intervals is a Poisson process, an average length of distortion set-up according to the considered gap length in the experiment, whereas the interval length results from the sequence length and number of intervals, which, for two gaps per sequence, are three—ahead of the first gap, in-between, and after the second gap.

## 4. Results and Discussion

The section comprises two parts. First, we present RMSE results; they illustrate the performance of each of the considered gap reconstruction methods. The second part is the interpretation of results, searching for the aspects of Mocap sequence that might affect the resulting performance.

### 4.1. Gap Reconstruction Efficiency

**Figure 7:**
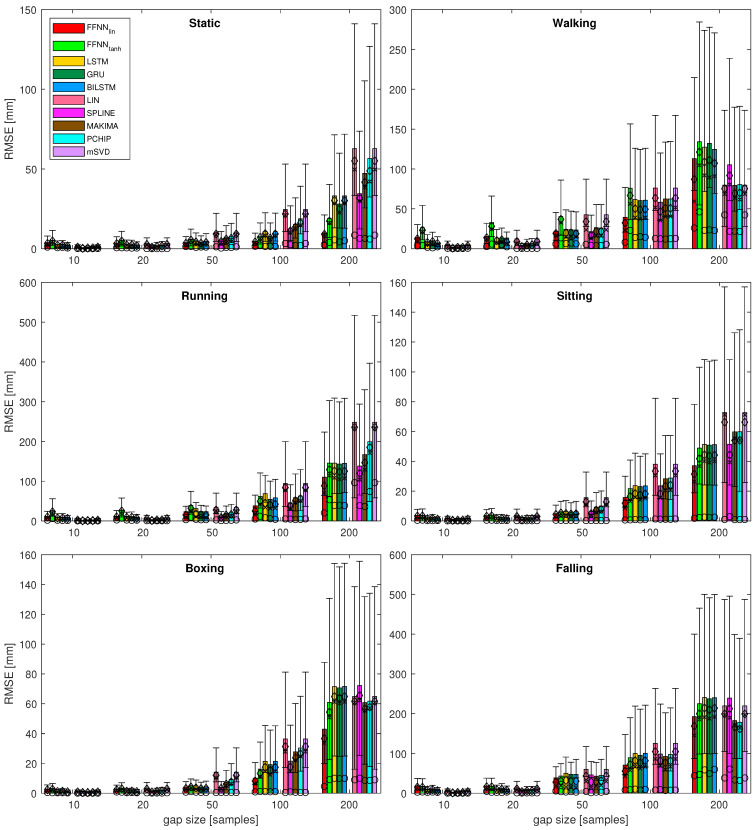
Results for most of the quality measures for all the test sequences. Bars denote RMSE; for $RMSE_{k}$: ⋄ denotes mean value, × denotes median, ∘ denotes mode, whiskers indicate IQR; standard deviation is not depicted here; dash-outlined areas are zoomed in Figure 8.

**Figure 8:**
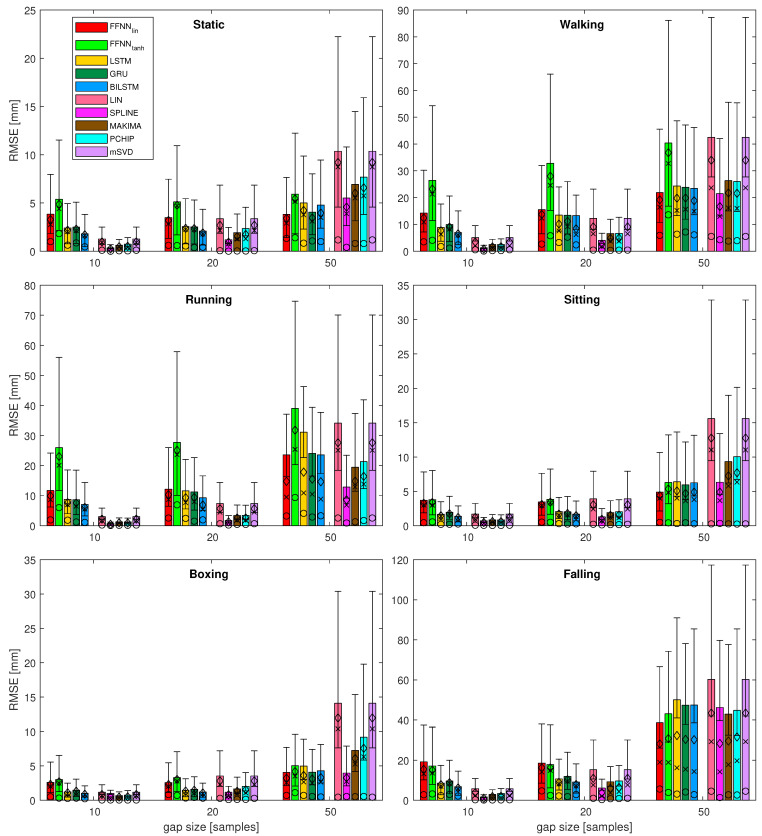
Results of the most of the quality measures for all the test sequences—zoomed variant for gaps 10, 20, and 50. Bars denote RMSE; for $RMSE_{k}$: ⋄ denotes mean value, × denotes median, ∘ denotes mode, whiskers indicate IQR; standard deviation is not depicted here.

The detailed numerical values are presented in Table 3 for the first sequence as an example. In the table, we also emphasize the best result for each measurement of gap size. Forclarity, the numerical outcomes of the experiment are only presented in this chapter with representative examples. To see the complete set of results in the tabular form, please refer to Appendix A. The complete results for the gap reconstruction are also demonstrated in a visual form in Figure 7. Additionally, the zoomed variant of the fragments of the plot (dash square annotated) for gaps 10–50 are presented in Figure 8.

The first observation, regarding the performance measures, is the fact that the results are very coherent, regardless of which measure was used. This is shown in Figure 7, where all the symbols coherently denote statistical descriptors scale. It is also clearly visible in the values emphasized in Table 3, where all measures but one (mode) indicate the same best (smallest) results. Hence, we can use a single quality measure; in our case, we assumed RMSE for further analysis.

Analyzing the results for several sequences, various observations regarding the performance of the considered methods can be noted. These are listed below:

- It can be seen that, for the short gaps, interpolation methods outperform any of the NN-based methods.
- For gaps that are 50 samples long, the results become less obvious and NN results are no worse or (usually) better than interpolation methods.
- Linear FFNN usually performed better than any other methods (including non-linear FFNN${}_{\tanh}$), for gaps of 50 samples or longer, for most of the sequences.
- In very rare cases of short-gap cases, RNNs performed better than FFNN${}_{\mathrm{lin}}$, but, in general, simpler FFNN${}_{\mathrm{lin}}$ outperformed more complex NN models.
- There are two situations when the FFNN${}_{\mathrm{lin}}$, performed no better or worse than interpolation methods (walking and falling). This occurred for sequences with larger monotonicity values in Table 2. They have also increased velocity/acceleration/jerk values; the ‘running’ sequence has similar values for these, but FFNN${}_{\mathrm{lin}}$ perform the best in this case, so the kinematic/dynamic parameters should not be considered.

Looking at the results of various NN architectures, it might be surprising that the sophisticated RNNs often returned worse results than relatively simple FFNN, especially for relatively long gaps. Conversely, one might expect that RNNs would outperform other methods, since they would be able to model longer-term dependencies in the motion. Presumably, the source of such a result is in the limited amount of training data, which, depending on the length of the source file, varies between hundreds and thousands of registered coordinates. Therefore, solvers are unable to find actually good values for a massive amount of parameters—see Table 4 for the formulas and numbers of learnable parameters for an exemplary case when input comprises 30 values—coordinates of four siblings and a parent at current and previous frames.

An obvious solution to such an issue would be increasing the training data. We could achieve this by employing very long recordings or by using numerous recordings. In the former, it would be difficult to achieve long enough recordings; the latter is different from the case which we try to address, where we only obtain a fresh mocap recording and reconstruct it with the minimal model given by FBM. Training the predictive model in advance with a massive amount of data is, of course, an interesting solution, but would cost the generality. For every marker configuration, a separate set of predicting NNs would need to be trained, so the result would only be practical for standardized body models.

**Figure 9:**
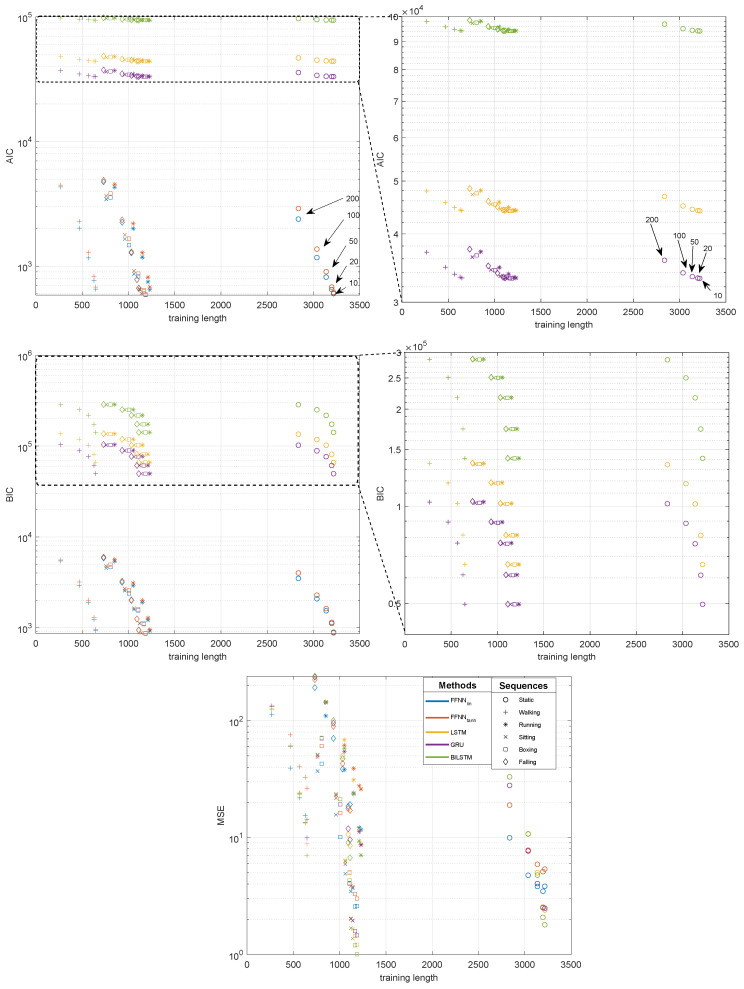
Influence of training sequence length on the quality of obtained results for NN methods: Akaike Information Criterion (AIC), Bayesian Information Criterion (BIC) and MSE.

Considering the length of the training sequences, its contribution to the final results seems far less important than other factors, at least within the range of considered cases. The analysis of its influence is illustrated in Figure 9. Since the MSE results are entangled, we employed two additional information criterions, Akaike Information Criterion (AIC), Bayesian Information Criterion (BIC), which disentangle the results by accounting for the number of trainable parameters. For every sequence and every NN model, we obtain a series of five results, which decrease, as the training sequence grows longer when we have shorter gaps (i.e., the annotated quintuple in the Figure). Analyzing the results in Figure 9, it is most convenient to observe this in the AIC/BIC plots since, for each model, the number of parameters remains the same (Table 4), so we can easily compare the results of the testing sequences. The zoomed versions (to the right) reveal differences at appropriate scales for the RNN results.

Lookng at the reults, we observe that, regardless the length of the training sequence, the MSE (AIC/BIC) of the NN model remains at the same order of magnitude—this is clearly visible in the Figure, where we have very similar values for each gap size for variable sequences (represented as different marker shapes) for each of the NN types (represented by a color). The most notable reduction in the error is probably observed with the increased sequence length, when the sequence (Seq. 1—static) is several folds longer than the others. However, we cannot observe this difference for shorter sequences in our data, with notably different lengths (e.g., walking—running). The quality of prediction could be likely improved if the recordings were longer, but, in everyday praxis, the length of the motion caputre sequences is only minutes, so one should not expect the results for RNN data to be notably improved compared to those for FFNN.

The observations hold for both FFNN models and all RNNs. These ambiguous outcomes confirm the results shown in [40], where the quality of results does not depend on the length of the training data in a straightforward way.

### 4.2. Motion Factors Affecting Performance

In this section, we try to identify the correlation in which features (parameters) of the input sequences relate to the performance of gap-filling methods. The results presented here are concise; we only present and discuss the most conclusive results. The complete tables containing correlation values for all gap sizes are presented in Appendix B.

Foremost, a generalized view into the correlation between gap-filling outcomes and input sequence characteristics is given in Table 5. It contains Pearson correlation coefficients (CC) between RMSE and input sequence characteristic parameters; the values are Pearson CCs, averaged across all the considered gap sizes. Additionally, for the interpretation of the results, in Table 6, we provide CCs between RMSE and the descriptive parameters for the whole sequences for all the test recordings.

Knowing that correlation, as a statistical measure, makes little sense for a sparse dataset, we treat it as a kind of measurement of co-linearity between the measures. However, for part of the parameters, the (high) correlation values are connected, with quite satisfactory low *p*-values; these are given in Appendix B.

Looking into the results in Table 5, we observe that all the considered sequence parameters are related, to some extent, to RMSE. However, for all the gap-filling methods, we identified two key parameters that have higher CCs than the others. These are acceleration and monotonicity, which seem to be promising candidate measures for describing the susceptibility of sequences to the employed reconstruction methods.

Regarding inter-parameter correlations in Table 6, we can observe that most of the measures are correlated with each other. This is expected, since kinematic/dynamic parameters are connected with the location of the markers over time, so values such as entropy, position standard deviation, velocity, acceleration, and jerk are correlated (for the derivatives, the smaller the difference in the derivative order, the higher the CCs).

On the other hand, the two less typical measures, monotonicity and complexity, are different; therefore, their correlation with the other measures is less predictable. Complexity appeared to have a notable negative correlation with most of the typical measures. Monotonicity, on the other hand, is more interesting. Since it is only moderately correlated with remaining measures, it still has quite a high CC, with RMSEs for all the gap reconstruction methods. Therefore, we can suppose this describes an aspect of the sequence that is independent of the other measures, which is related to susceptibility to the gap reconstruction procedures.

## 5. Summary

In this article, we addressed the issue of filling the gaps that occurred in the mocap signal. We considered this to be a regressive problem and reviewed the results of several NN-based regressors, which were compared with several interpolation and low-rank matrix completion (mSVD) methods.

Generally, in the case of short gaps, the interpolation methods returned the best results, but since the gaps became longer, part of the NNs gained an advantage. We reviewed five variants of neural networks. Surprisingly, the tests revealed that simple linear FFNNs, using momentary (current and previous sample) and local (from neighboring markers) coordinates as input data, outperformed quite advanced recurrent NNs for the longer gaps. For the shorter gaps, RNNs offered better results, but all the NNs were outperformed by interpolations. The boundary between ’long’ and ’short’ terms are gaps of 50 samples long. Finally, we were able to identify which factors of the input mocap sequence influence the reconstruction errors.

The approach to the NNs given here does not incorporate skeletal information. Instead, the kinematic structure is based on the FBM framework and all the predictions are performed with the local data, as obtained from FBM. Currently, none of the analyzed approaches considered body constraints such as limb length or size, but we can easily obtain such information from the FBM model. We plan to apply this as an additional processing stage in the future. In the future, we plan to test more sophisticated NN architectures, such as combined LSTM convolution, or averaged multiregressions.

## Figures and Tables

**Table 1:** List of mocap sequence scenarios used for the testing.

No.	Name	Scenario	Duration	Difficulty
1	Static	Actor stands in the middle of scene, looking around and shifting from one foot to another, freely swinging arms	32 s	varied motions
2	Walking	Actor stands still at the edge of the scene, then walks straight for 6 m, then stands still	7 s	low dynamics, easy
3	Running	Actor stands in the middle of scene, then goes backwards to the edge of the scene and runs for 6 m, then goes backwards to the middle of the scene	16 s	moderate dynamics
4	Sitting	Actor stands in the middle of scene, then sits on a stool, and, after a few seconds, stands again	15 s	occlusions
5	Boxing	Actor stands in the middle of scene, and performs some fast boxing punches	14 s	high dynamics
6	Falling	Actor stands on 0.5 m elevation in the middle of scene, the walks to edge of platform, then falls on the mattress, lies for 2 s and stands	16 s	high dynamics, occlusions

**Table 2:** Input sequence characteristics.

No	Entropy (H(X))	Stddev ($\sigma_{X}$)	Velocity ($\frac{\partial X}{\partial t}$)	Acc. ($\frac{\partial^{2}X}{\partial t^{2}}$)	Jerk ($\frac{\partial^{3}X}{\partial t^{3}}$)	Monotonicity	Complexity
	[Bits/Mark.]	[mm/Coordinate]	[m/s/Mark.]	$\left[m/s^{2}/\mathrm{Mark}.\right]$	$\left[m/s^{3}/\mathrm{Mark}.\right]$	[-]	[-]
1	12.697	129.705	0.208	1.561	64.817	0.352	0.027
2	13.943	941.123	0.773	6.476	829.271	0.582	0.000
3	15.710	982.342	0.895	6.176	643.337	0.379	0.001
4	10.231	135.356	0.190	2.863	452.142	0.347	0.016
5	11.356	121.094	0.259	3.557	507.975	0.323	0.023
6	14.152	601.140	0.589	6.703	799.039	0.745	0.007

**Table 3:** Quality measures for the static (No. 1) sequence.

Len		FFNN${}_{\mathrm{lin}}$	FFNN${}_{\tanh}$	LSTM	GRU	BILSTM	LIN	SPLINE	MAKIMA	PCHIP	mSVD
10	RMSE	3.830	5.375	2.410	2.494	1.801	1.267	**0.348**	0.610	0.737	1.267
	mean (${\mathrm{RMSE}}_{k}$)	3.280	4.869	2.175	2.290	1.708	0.971	**0.243**	0.468	0.512	0.971
	median (${\mathrm{RMSE}}_{k}$)	2.746	4.399	2.035	2.120	1.614	0.893	**0.205**	0.406	0.391	0.893
	mode (${\mathrm{RMSE}}_{k}$)	0.993	1.821	0.626	0.861	0.455	0.099	**0.000**	0.045	0.036	0.099
	stddev (${\mathrm{RMSE}}_{k}$)	1.893	2.209	0.939	0.989	0.573	0.695	**0.216**	0.336	0.458	0.695
	iqr (${\mathrm{RMSE}}_{k}$)	2.123	2.905	0.881	0.901	0.684	0.692	**0.235**	0.370	0.434	0.692
20	RMSE	3.474	5.114	2.559	2.527	2.082	3.366	**1.191**	1.914	2.354	3.366
	mean (${\mathrm{RMSE}}_{k}$)	3.187	4.775	2.371	2.351	1.903	2.694	**0.933**	1.525	1.738	2.694
	median (${\mathrm{RMSE}}_{k}$)	2.828	4.709	2.274	2.235	1.779	2.147	**0.764**	1.251	1.287	2.147
	mode (${\mathrm{RMSE}}_{k}$)	0.605	0.584	0.540	0.381	0.415	0.052	**0.005**	0.026	0.023	0.052
	stddev (${\mathrm{RMSE}}_{k}$)	1.442	1.871	0.891	0.898	0.826	1.831	**0.664**	1.045	1.483	1.831
	iqr (${\mathrm{RMSE}}_{k}$)	1.841	2.394	1.103	1.013	0.813	1.983	**0.866**	1.173	1.437	1.983
50	RMSE	**3.813**	5.910	5.001	4.041	4.777	10.363	5.517	6.928	7.677	10.363
	mean (${\mathrm{RMSE}}_{k}$)	**3.401**	5.434	4.233	3.445	3.958	9.207	4.572	6.027	6.573	9.207
	median (${\mathrm{RMSE}}_{k}$)	**2.906**	5.154	3.776	3.118	3.496	8.733	3.888	5.512	5.733	8.733
	mode (${\mathrm{RMSE}}_{k}$)	1.326	1.393	0.831	1.066	1.000	1.169	**0.400**	0.800	0.793	1.169
	stddev (${\mathrm{RMSE}}_{k}$)	**1.688**	2.168	2.430	1.921	2.448	4.464	2.852	3.174	3.764	4.464
	iqr (${\mathrm{RMSE}}_{k}$)	**1.421**	2.216	2.169	1.642	2.282	6.078	2.418	3.770	4.373	6.078
100	RMSE	**4.759**	7.805	10.798	7.678	10.716	24.634	12.548	15.231	18.746	24.634
	mean (${\mathrm{RMSE}}_{k}$)	**4.233**	7.134	9.460	6.721	9.302	21.812	11.236	13.587	16.108	21.812
	median (${\mathrm{RMSE}}_{k}$)	**3.658**	6.329	8.333	5.953	8.198	21.129	10.345	12.875	14.785	21.129
	mode (${\mathrm{RMSE}}_{k}$)	1.517	2.252	**1.377**	1.465	1.400	3.266	2.546	1.986	1.937	3.266
	stddev (${\mathrm{RMSE}}_{k}$)	**2.132**	3.143	5.114	3.692	5.230	11.305	5.472	6.825	9.556	11.305
	iqr (${\mathrm{RMSE}}_{k}$)	**2.215**	3.473	5.650	4.217	5.700	14.536	6.850	8.029	11.019	14.536
200	RMSE	**9.959**	18.970	33.147	27.987	33.104	62.786	34.481	47.259	56.570	62.786
	mean (${\mathrm{RMSE}}_{k}$)	**9.062**	17.303	30.204	24.837	30.135	55.099	31.616	41.676	48.789	55.099
	median (${\mathrm{RMSE}}_{k}$)	**8.683**	16.200	28.352	22.655	28.462	49.641	29.914	38.410	42.155	49.641
	mode (${\mathrm{RMSE}}_{k}$)	**2.404**	3.973	5.523	4.263	5.010	8.510	6.518	6.459	6.033	8.510
	stddev (${\mathrm{RMSE}}_{k}$)	**4.013**	7.631	13.450	12.743	13.503	29.934	13.511	22.022	28.463	29.934
	iqr (${\mathrm{RMSE}}_{k}$)	**5.084**	9.413	18.231	16.895	18.436	48.864	17.125	36.315	46.222	48.864

**Table 4:** List of mocap sequence scenarios used for the testing.

NN Type	Number of Learnable Parameters	Value for Exemplary Case
FFNN:	hiddenLayerSize×inputvectorSize+hiddenLayerSize	275
	+3×hiddenLayerSize+3	
LSTM:	4×hiddenRecurrentNeurons×inputvectorSize	22,023
	+4×hiddenRecurrentNeurons×hiddenRecurrentNeurons	
	+4×hiddenRecurrentNeurons	
	+3×hiddenRecurrentNeurons+3	
GRU:	3×hiddenRecurrentNeurons×inputvectorSize	16,563
	+3×hiddenRecurrentNeurons×hiddenRecurrentNeurons	
	+3×hiddenRecurrentNeurons	
	+3×hiddenRecurrentNeurons+3	
BILSTM:	8×hiddenRecurrentNeurons×inputvectorSize	47,043
	+8×hiddenRecurrentNeurons×hiddenRecurrentNeurons	
	+8×hiddenRecurrentNeurons	
	+3×2×hiddenRecurrentNeurons+3	

**Table 5:** Correlation between RMSE and sequence parameters (averaged for all gap sizes).

	FFNN${}_{\mathrm{lin}}$	FFNN${}_{\tanh}$	LSTM	GRU	BILSTM	LIN	SPLINE	MAKIMA	PCHIP	mSVD
**Entropy**	0.708	0.793	0.775	0.736	0.735	0.680	0.486	0.624	0.630	0.680
**Stddev**	0.741	0.892	0.805	0.781	0.778	0.706	0.517	0.653	0.631	0.706
**Velocity**	0.744	0.886	0.813	0.784	0.781	0.713	0.521	0.656	0.640	0.713
**Acceleration**	0.905	0.912	0.903	0.907	0.890	0.854	0.791	0.844	0.818	0.854
**Jerk**	0.803	0.794	0.777	0.799	0.779	0.753	0.758	0.763	0.725	0.753
**Monotonicity**	0.900	0.713	0.798	0.847	0.819	0.824	0.926	0.888	0.862	0.824
**Complexity**	−0.779	−0.886	−0.815	−0.804	−0.794	−0.742	−0.589	−0.702	−0.670	−0.742

**Table 6:** Correlation between sequence parameters.

	Entropy	Stddev	Velocity	Acceleration	Jerk	Monotonicity	Complexity
**Entropy**	1.000	0.869	0.898	0.730	0.459	0.465	−0.712
**Stddev**	0.869	1.000	0.992	0.879	0.732	0.501	−0.949
**Velocity**	0.898	0.992	1.000	0.890	0.731	0.477	−0.929
**Acceleration**	0.730	0.879	0.890	1.000	0.941	0.735	−0.913
**Jerk**	0.459	0.732	0.731	0.941	1.000	0.695	−0.847
**Monotonicity**	0.465	0.501	0.477	0.735	0.695	1.000	−0.560
**Complexity**	−0.712	−0.949	−0.929	−0.913	−0.847	−0.560	1.000
				*p*-values			
**Entropy**	1.000	0.025	0.015	0.100	0.360	0.353	0.112
**Stddev**	0.025	1.000	0.000	0.021	0.098	0.311	0.004
**Velocity**	0.015	0.000	1.000	0.017	0.099	0.338	0.007
**Acceleration**	0.100	0.021	0.017	1.000	0.005	0.096	0.011
**Jerk**	0.360	0.098	0.099	0.005	1.000	0.125	0.033
**Monotonicity**	0.353	0.311	0.338	0.096	0.125	1.000	0.248
**Complexity**	0.112	0.004	0.007	0.011	0.033	0.248	1.000

## Data Availability

The motion capture sequences are provided as Supplementary Files accompanying the article.

## Supplementary Materials

The following are available at https://www.mdpi.com/1424-8220/21/18/6115/s1, The motion capture sequences.

## Abbreviations

The following abbreviations are used in this manuscript:

BILSTM	bidirectional LSTM
CC	correlation coefficient
FC	fully connected
FBM	functional body mesh
FFNN	feed forward neural network
GRU	gated recurrent unit
HML	Human Motion Laboratory
IK	inverse kinematics
KF	Kalman filter
LS	least squares
LSTM	long-short term memory
Mocap	MOtion CAPture
MSE	Mean Square Error
NARX-NN	nonlinear autoregressive exogenous neural network
NaN	not a number
NN	neural network
OMC	optical motion capture
PCA	principal component analysis
PJAIT	Polish-Japanese Academy of Information Technology
RMSE	root mean squared error
RNN	recurrent neural network
STDDEV	standard deviation
SVD	singular value decomposition

## Appendix A. Performance Results for All Sequences

**Table A1:** Quality measures for the walking (No. 2) sequence.

Len		FFNN${}_{\mathrm{lin}}$	FFNN${}_{\tanh}$	LSTM	GRU	BILSTM	LIN	SPLINE	MAKIMA	PCHIP	mSVD
10	RMSE	14.222	26.428	8.844	9.932	7.004	5.088	1.287	2.464	2.507	5.088
	mean (${\mathrm{RMSE}}_{k}$)	12.398	23.213	7.659	9.014	6.495	3.442	0.810	1.621	1.697	3.442
	median (${\mathrm{RMSE}}_{k}$)	10.865	21.290	6.327	8.262	5.956	2.051	0.511	1.087	1.180	2.051
	mode (${\mathrm{RMSE}}_{k}$)	3.499	4.068	1.755	1.140	2.344	0.536	0.056	0.237	0.239	0.536
	stddev (${\mathrm{RMSE}}_{k}$)	6.930	12.645	4.634	4.744	3.371	3.505	0.938	1.773	1.788	3.505
	iqr (${\mathrm{RMSE}}_{k}$)	8.986	12.914	3.644	4.297	3.444	3.180	0.652	1.293	1.334	3.180
20	RMSE	15.490	32.802	13.491	13.382	13.303	12.274	4.031	6.590	6.619	12.274
	mean (${\mathrm{RMSE}}_{k}$)	13.743	27.978	10.155	11.171	8.396	9.071	2.591	4.798	4.904	9.071
	median (${\mathrm{RMSE}}_{k}$)	12.334	24.575	7.568	9.116	6.209	6.508	1.823	3.767	3.728	6.508
	mode (${\mathrm{RMSE}}_{k}$)	2.654	5.774	3.242	5.247	2.352	0.401	0.314	0.316	0.382	0.401
	stddev (${\mathrm{RMSE}}_{k}$)	6.723	16.042	8.161	6.609	8.827	8.020	2.828	4.290	4.175	8.020
	iqr (${\mathrm{RMSE}}_{k}$)	7.454	15.726	4.545	5.667	2.491	6.791	1.571	3.308	3.921	6.791
50	RMSE	21.907	40.375	24.343	23.833	23.434	42.517	21.474	26.332	25.995	42.517
	mean (${\mathrm{RMSE}}_{k}$)	19.168	36.769	19.788	19.867	18.831	33.944	16.673	21.757	21.607	33.944
	median (${\mathrm{RMSE}}_{k}$)	16.432	32.752	15.196	15.655	14.926	23.652	12.952	16.134	15.996	23.652
	mode (${\mathrm{RMSE}}_{k}$)	5.905	13.574	6.336	7.173	6.100	5.500	4.293	3.782	3.921	5.500
	stddev (${\mathrm{RMSE}}_{k}$)	10.486	16.289	13.174	12.408	13.037	25.484	12.659	14.438	13.993	25.484
	iqr (${\mathrm{RMSE}}_{k}$)	12.421	22.207	13.308	11.413	12.903	29.918	12.189	17.991	18.129	29.918
100	RMSE	39.346	75.817	61.641	60.420	60.823	76.058	58.357	62.302	62.419	76.058
	mean (${\mathrm{RMSE}}_{k}$)	32.287	66.701	50.195	49.019	49.453	63.445	46.476	50.803	50.693	63.445
	median (${\mathrm{RMSE}}_{k}$)	23.318	56.329	38.960	37.001	39.074	51.683	35.447	40.065	40.418	51.683
	mode (${\mathrm{RMSE}}_{k}$)	8.122	22.940	14.125	15.094	14.334	12.943	12.407	12.074	12.493	12.943
	stddev (${\mathrm{RMSE}}_{k}$)	22.397	35.709	35.107	34.707	34.685	41.564	34.503	35.371	35.700	41.564
	iqr (${\mathrm{RMSE}}_{k}$)	18.933	41.446	39.727	40.427	40.813	63.062	39.062	49.784	50.440	63.062
200	RMSE	112.933	134.121	127.416	132.150	124.566	79.741	105.237	79.407	80.457	79.741
	mean (${\mathrm{RMSE}}_{k}$)	87.084	121.229	108.733	111.164	107.192	75.307	91.826	69.585	70.031	75.307
	median (${\mathrm{RMSE}}_{k}$)	59.288	104.710	91.987	89.523	91.019	68.567	80.427	63.559	61.704	68.567
	mode (${\mathrm{RMSE}}_{k}$)	26.007	46.150	23.032	23.675	22.813	42.408	21.841	21.984	21.602	42.408
	stddev (${\mathrm{RMSE}}_{k}$)	71.160	57.197	66.944	71.470	63.693	26.502	53.401	39.296	40.746	26.502
	iqr (${\mathrm{RMSE}}_{k}$)	61.864	71.116	90.839	90.285	90.685	42.057	88.500	65.862	66.873	42.057

**Table A2:** Quality measures for the running (No. 3) sequence.

Len		FFNN${}_{\mathrm{lin}}$	FFNN${}_{\tanh}$	LSTM	GRU	BILSTM	LIN	SPLINE	MAKIMA	PCHIP	mSVD
10	RMSE	11.702	25.988	8.748	8.666	7.066	3.001	0.701	1.291	1.259	3.001
	mean (${\mathrm{RMSE}}_{k}$)	9.939	23.049	7.675	7.581	6.105	2.221	0.476	0.985	0.942	2.221
	median (${\mathrm{RMSE}}_{k}$)	8.661	20.122	6.973	6.485	5.540	1.743	0.346	0.831	0.720	1.743
	mode (${\mathrm{RMSE}}_{k}$)	1.933	6.022	1.838	1.236	1.106	0.234	0.079	0.149	0.151	0.234
	stddev (${\mathrm{RMSE}}_{k}$)	5.919	11.837	4.214	4.245	3.797	1.714	0.439	0.692	0.691	1.714
	iqr (${\mathrm{RMSE}}_{k}$)	7.005	15.692	5.106	4.850	3.513	1.835	0.286	0.835	0.799	1.835
20	RMSE	12.141	27.729	11.594	11.232	9.321	7.397	1.742	3.401	3.439	7.397
	mean (${\mathrm{RMSE}}_{k}$)	10.331	25.124	9.324	9.440	6.919	5.676	1.274	2.601	2.589	5.676
	median (${\mathrm{RMSE}}_{k}$)	8.695	23.641	7.664	7.948	5.424	4.496	0.968	1.988	1.853	4.496
	mode (${\mathrm{RMSE}}_{k}$)	2.547	6.946	2.438	1.512	1.953	0.661	0.237	0.453	0.438	0.661
	stddev (${\mathrm{RMSE}}_{k}$)	6.215	11.425	6.552	5.753	5.787	4.017	1.010	1.889	2.021	4.017
	iqr (${\mathrm{RMSE}}_{k}$)	8.168	12.490	4.481	5.442	3.111	3.995	1.017	2.154	2.061	3.995
50	RMSE	23.573	39.084	31.147	24.057	23.597	34.144	12.857	19.473	21.328	34.144
	mean (${\mathrm{RMSE}}_{k}$)	14.767	31.801	17.835	15.504	14.637	27.624	8.608	14.842	16.431	27.624
	median (${\mathrm{RMSE}}_{k}$)	9.523	25.412	10.904	10.501	8.853	25.122	6.834	12.894	13.844	25.122
	mode (${\mathrm{RMSE}}_{k}$)	3.229	9.379	4.119	2.888	3.306	2.559	0.896	1.291	1.737	2.559
	stddev (${\mathrm{RMSE}}_{k}$)	18.345	22.596	25.456	18.049	18.231	18.865	8.914	11.837	12.760	18.865
	iqr (${\mathrm{RMSE}}_{k}$)	6.432	16.838	6.719	7.811	7.903	20.224	6.920	9.883	11.590	20.224
100	RMSE	38.173	61.656	68.606	54.639	58.223	94.347	45.740	58.606	62.724	94.347
	mean (${\mathrm{RMSE}}_{k}$)	25.165	49.288	44.780	40.344	42.251	83.854	37.303	51.072	55.958	83.854
	median (${\mathrm{RMSE}}_{k}$)	18.493	41.944	33.811	31.168	32.177	77.220	32.103	46.438	50.903	77.220
	mode (${\mathrm{RMSE}}_{k}$)	4.901	11.780	8.178	5.555	4.181	4.989	4.549	3.554	3.884	4.989
	stddev (${\mathrm{RMSE}}_{k}$)	27.594	35.231	50.041	35.158	38.271	41.350	25.286	27.575	27.272	41.350
	iqr (${\mathrm{RMSE}}_{k}$)	13.060	29.863	24.844	24.922	25.449	47.512	25.816	26.432	29.725	47.512
200	RMSE	110.196	145.641	145.387	143.360	145.050	248.552	138.231	167.249	199.417	248.552
	mean (${\mathrm{RMSE}}_{k}$)	88.708	129.262	125.767	123.634	125.213	235.787	119.848	146.780	185.085	235.787
	median (${\mathrm{RMSE}}_{k}$)	70.845	113.902	108.387	105.181	107.987	233.618	103.952	128.657	171.109	233.618
	mode (${\mathrm{RMSE}}_{k}$)	20.092	53.434	39.113	39.722	38.728	96.336	38.444	36.027	74.145	96.336
	stddev (${\mathrm{RMSE}}_{k}$)	63.969	65.135	70.990	70.695	71.285	73.293	66.963	77.021	70.628	73.293
	iqr (${\mathrm{RMSE}}_{k}$)	67.200	73.343	87.747	82.080	89.947	77.986	83.010	64.869	47.085	77.986

**Table A3:** Quality measures for the sitting (No. 4) sequence.

Len		FFNN${}_{\mathrm{lin}}$	FFNN${}_{\tanh}$	LSTM	GRU	BILSTM	LIN	SPLINE	MAKIMA	PCHIP	mSVD
10	RMSE	3.701	3.792	1.664	1.954	1.373	1.697	0.711	0.841	0.839	1.697
	mean (${\mathrm{RMSE}}_{k}$)	3.272	3.386	1.463	1.737	1.210	1.218	0.478	0.617	0.606	1.218
	median (${\mathrm{RMSE}}_{k}$)	2.996	2.987	1.351	1.682	1.108	0.948	0.339	0.475	0.429	0.948
	mode (${\mathrm{RMSE}}_{k}$)	0.437	0.558	0.197	0.212	0.249	0.072	0.059	0.041	0.043	0.072
	stddev (${\mathrm{RMSE}}_{k}$)	1.896	1.767	0.806	0.846	0.642	1.094	0.483	0.530	0.537	1.094
	iqr (${\mathrm{RMSE}}_{k}$)	2.282	2.025	0.991	1.301	0.702	1.049	0.260	0.467	0.480	1.049
20	RMSE	3.464	3.829	2.060	2.025	1.688	3.902	1.285	1.904	2.029	3.902
	mean (${\mathrm{RMSE}}_{k}$)	3.106	3.429	1.708	1.797	1.475	3.057	0.942	1.515	1.559	3.057
	median (${\mathrm{RMSE}}_{k}$)	2.911	3.319	1.519	1.572	1.318	2.434	0.739	1.230	1.169	2.434
	mode (${\mathrm{RMSE}}_{k}$)	0.522	0.497	0.300	0.240	0.271	0.211	0.126	0.155	0.161	0.211
	stddev (${\mathrm{RMSE}}_{k}$)	1.577	1.750	1.122	0.962	0.812	2.415	0.838	1.153	1.311	2.415
	iqr (${\mathrm{RMSE}}_{k}$)	2.233	2.263	1.038	1.069	0.934	2.762	0.781	0.979	0.995	2.762
20	RMSE	4.901	6.291	6.392	5.952	6.255	15.596	6.334	9.332	10.056	15.596
	mean (${\mathrm{RMSE}}_{k}$)	4.383	5.355	5.064	4.697	4.895	12.767	4.902	7.260	7.710	12.767
	median (${\mathrm{RMSE}}_{k}$)	3.982	4.831	4.007	3.623	3.803	11.036	3.652	5.788	6.343	11.036
	mode (${\mathrm{RMSE}}_{k}$)	0.482	0.417	0.313	0.422	0.277	0.267	0.332	0.267	0.240	0.267
	stddev (${\mathrm{RMSE}}_{k}$)	2.276	3.254	3.793	3.568	3.778	8.741	3.880	5.667	6.265	8.741
	iqr (${\mathrm{RMSE}}_{k}$)	2.978	3.833	5.160	4.098	4.999	11.116	5.269	6.546	6.801	11.116
20	RMSE	15.716	21.780	23.727	23.023	23.575	38.083	23.547	28.358	28.813	38.083
	mean (${\mathrm{RMSE}}_{k}$)	11.904	16.468	18.440	17.539	18.222	33.439	18.245	23.435	24.033	33.439
	median (${\mathrm{RMSE}}_{k}$)	8.596	13.132	15.903	14.109	15.147	30.517	15.467	20.365	20.691	30.517
	mode (${\mathrm{RMSE}}_{k}$)	0.643	0.711	0.927	0.743	0.950	1.324	1.170	1.139	1.121	1.324
	stddev (${\mathrm{RMSE}}_{k}$)	9.980	13.839	14.495	14.484	14.524	17.840	14.459	15.569	15.542	17.840
	iqr (${\mathrm{RMSE}}_{k}$)	7.816	11.087	13.380	12.476	13.201	23.419	13.054	15.405	14.280	23.419
20	RMSE	37.101	48.909	51.388	50.842	51.274	72.745	51.478	59.839	59.857	72.745
	mean (${\mathrm{RMSE}}_{k}$)	31.439	41.811	44.331	43.711	44.219	66.280	44.321	54.030	54.156	66.280
	median (${\mathrm{RMSE}}_{k}$)	26.422	36.792	40.178	39.257	40.099	71.201	39.395	55.235	54.311	71.201
	mode (${\mathrm{RMSE}}_{k}$)	1.783	2.342	2.592	2.372	2.558	0.972	2.819	0.875	0.912	0.972
	stddev (${\mathrm{RMSE}}_{k}$)	20.198	25.924	26.514	26.496	26.480	30.443	26.659	26.183	26.001	30.443
	iqr (${\mathrm{RMSE}}_{k}$)	22.947	30.188	29.510	29.617	29.241	37.209	29.316	29.572	28.094	37.209

**Table A4:** Quality measures for the boxing (No. 5) sequence.

Len		FFNN${}_{\mathrm{lin}}$	FFNN${}_{\tanh}$	LSTM	GRU	BILSTM	LIN	SPLINE	MAKIMA	PCHIP	mSVD
10	RMSE	2.603	3.006	1.217	1.467	1.008	1.175	0.986	0.668	0.735	1.175
	mean (${\mathrm{RMSE}}_{k}$)	2.321	2.697	1.087	1.316	0.885	0.848	0.484	0.461	0.507	0.848
	median (${\mathrm{RMSE}}_{k}$)	2.036	2.476	1.001	1.173	0.783	0.666	0.276	0.317	0.322	0.666
	mode (${\mathrm{RMSE}}_{k}$)	0.505	0.309	0.270	0.303	0.218	0.036	0.043	0.035	0.034	0.036
	stddev (${\mathrm{RMSE}}_{k}$)	1.174	1.354	0.521	0.613	0.456	0.712	0.765	0.420	0.473	0.712
	iqr (${\mathrm{RMSE}}_{k}$)	1.449	1.769	0.504	0.705	0.542	0.709	0.307	0.341	0.490	0.709
20	RMSE	2.581	3.298	1.446	1.591	1.200	3.534	1.157	1.648	2.021	3.534
	mean (${\mathrm{RMSE}}_{k}$)	2.295	3.030	1.341	1.458	1.070	2.818	0.797	1.309	1.519	2.818
	median (${\mathrm{RMSE}}_{k}$)	2.022	2.780	1.242	1.353	0.934	2.282	0.608	0.983	1.071	2.282
	mode (${\mathrm{RMSE}}_{k}$)	0.826	0.700	0.326	0.402	0.303	0.273	0.106	0.125	0.126	0.273
	stddev (${\mathrm{RMSE}}_{k}$)	1.161	1.308	0.541	0.606	0.549	1.965	0.819	0.930	1.249	1.965
	iqr (${\mathrm{RMSE}}_{k}$)	1.415	1.704	0.736	0.732	0.494	2.153	0.491	1.038	1.333	2.153
50	RMSE	4.045	5.038	4.965	4.067	4.295	14.095	3.956	7.248	9.171	14.095
	mean (${\mathrm{RMSE}}_{k}$)	3.211	4.183	3.609	3.109	3.306	11.957	3.262	6.083	7.562	11.957
	median (${\mathrm{RMSE}}_{k}$)	2.546	3.503	2.661	2.500	2.634	10.384	2.736	5.271	6.318	10.384
	mode (${\mathrm{RMSE}}_{k}$)	0.699	1.102	0.699	0.538	0.480	0.444	0.542	0.513	0.546	0.444
	stddev (${\mathrm{RMSE}}_{k}$)	2.460	2.788	3.404	2.614	2.747	7.236	2.235	3.802	4.994	7.236
	iqr (${\mathrm{RMSE}}_{k}$)	1.743	1.595	1.968	1.540	2.062	9.821	2.059	5.074	7.302	9.821
100	RMSE	10.134	16.216	21.424	19.275	21.386	36.436	21.538	27.723	30.374	36.436
	mean (${\mathrm{RMSE}}_{k}$)	8.175	13.241	17.438	15.384	17.357	31.336	17.608	23.779	26.421	31.336
	median (${\mathrm{RMSE}}_{k}$)	6.398	11.337	14.702	12.285	14.627	27.834	14.823	22.008	24.825	27.834
	mode (${\mathrm{RMSE}}_{k}$)	0.864	1.156	1.085	0.973	1.090	0.514	0.912	0.632	0.490	0.514
	stddev (${\mathrm{RMSE}}_{k}$)	5.837	9.220	12.123	11.372	12.169	18.465	12.075	14.128	14.876	18.465
	iqr (${\mathrm{RMSE}}_{k}$)	6.261	12.033	16.415	16.672	16.414	25.577	16.361	18.637	19.008	25.577
200	RMSE	42.833	60.847	71.465	70.625	71.514	64.829	72.201	60.721	61.704	64.829
	mean (${\mathrm{RMSE}}_{k}$)	36.693	54.330	64.743	63.732	64.805	61.507	65.477	56.493	57.666	61.507
	median (${\mathrm{RMSE}}_{k}$)	33.631	50.764	61.017	60.170	61.057	60.782	62.218	55.492	57.030	60.782
	mode (${\mathrm{RMSE}}_{k}$)	4.592	9.116	10.042	9.788	9.974	8.998	10.077	8.616	8.609	8.998
	stddev (${\mathrm{RMSE}}_{k}$)	21.768	26.954	29.620	29.819	29.609	20.171	29.798	22.097	21.740	20.171
	iqr (${\mathrm{RMSE}}_{k}$)	21.992	31.408	36.039	35.205	36.075	24.945	36.485	29.814	28.505	24.945

**Table A5:** Quality measures for the falling (No. 6) sequence.

Len		FFNN${}_{\mathrm{lin}}$	FFNN${}_{\tanh}$	LSTM	GRU	BILSTM	LIN	SPLINE	MAKIMA	PCHIP	mSVD
10	RMSE	19.193	17.106	8.537	9.585	6.720	5.763	1.601	2.872	3.365	5.763
	mean (${\mathrm{RMSE}}_{k}$)	15.455	15.022	7.818	8.772	6.166	3.827	0.994	1.851	1.968	3.827
	median (${\mathrm{RMSE}}_{k}$)	13.186	13.571	6.947	8.341	5.616	2.359	0.618	1.107	1.145	2.359
	mode (${\mathrm{RMSE}}_{k}$)	2.760	3.139	2.310	2.880	2.110	0.244	0.105	0.145	0.149	0.244
	stddev (${\mathrm{RMSE}}_{k}$)	11.270	8.163	3.494	3.852	2.555	4.023	1.138	2.039	2.551	4.023
	iqr (${\mathrm{RMSE}}_{k}$)	9.203	10.174	3.101	4.009	3.520	3.723	0.789	1.795	1.813	3.723
20	RMSE	18.496	17.762	10.664	11.914	9.057	15.278	6.073	9.213	9.596	15.278
	mean (${\mathrm{RMSE}}_{k}$)	16.206	16.199	8.940	10.261	7.897	10.937	3.694	5.981	6.392	10.937
	median (${\mathrm{RMSE}}_{k}$)	14.108	14.897	8.130	9.530	7.106	7.613	2.089	3.687	4.319	7.613
	mode (${\mathrm{RMSE}}_{k}$)	4.659	2.388	2.143	1.822	2.821	0.953	0.339	0.756	0.832	0.953
	stddev (${\mathrm{RMSE}}_{k}$)	8.511	7.184	6.211	5.915	4.455	9.596	4.383	6.169	6.567	9.596
	iqr (${\mathrm{RMSE}}_{k}$)	9.496	8.219	4.321	5.520	3.642	10.133	3.402	5.298	5.154	10.133
50	RMSE	38.618	43.058	50.077	47.367	47.474	60.232	46.220	42.945	44.782	60.232
	mean (${\mathrm{RMSE}}_{k}$)	28.149	30.795	32.292	30.356	30.213	43.423	28.314	29.543	31.603	43.423
	median (${\mathrm{RMSE}}_{k}$)	18.927	18.873	16.214	15.491	14.312	29.262	14.172	17.724	19.705	29.262
	mode (${\mathrm{RMSE}}_{k}$)	5.585	3.883	3.061	4.112	2.789	4.507	1.345	2.710	2.615	4.507
	stddev (${\mathrm{RMSE}}_{k}$)	25.916	29.395	37.233	35.345	35.587	42.053	35.660	31.333	31.914	42.053
	iqr (${\mathrm{RMSE}}_{k}$)	15.417	17.126	31.871	21.094	29.043	38.828	27.009	24.239	28.168	38.828
100	RMSE	70.671	89.650	100.005	95.523	100.282	125.495	98.770	92.878	97.573	125.495
	mean (${\mathrm{RMSE}}_{k}$)	55.641	72.172	81.983	76.503	81.814	104.667	81.794	76.620	81.261	104.667
	median (${\mathrm{RMSE}}_{k}$)	42.728	57.532	66.468	62.277	66.311	86.119	68.990	59.710	66.757	86.119
	mode (${\mathrm{RMSE}}_{k}$)	7.967	8.688	10.247	7.912	9.749	7.809	9.146	6.892	7.449	7.809
	stddev (${\mathrm{RMSE}}_{k}$)	43.593	53.283	57.286	57.268	58.033	69.796	55.712	52.857	54.185	69.796
	iqr (${\mathrm{RMSE}}_{k}$)	52.533	72.029	82.980	85.218	86.618	85.060	92.529	71.859	75.211	85.060
200	RMSE	192.371	224.989	240.459	237.068	240.104	219.332	238.962	182.390	177.973	219.332
	mean (${\mathrm{RMSE}}_{k}$)	168.542	199.701	214.118	209.626	213.731	198.998	212.497	165.908	161.330	198.998
	median (${\mathrm{RMSE}}_{k}$)	145.399	185.636	190.954	187.446	191.565	196.704	189.560	169.458	163.676	196.704
	mode (${\mathrm{RMSE}}_{k}$)	43.924	47.226	58.636	49.156	60.128	38.386	60.706	33.396	32.207	38.386
	stddev (${\mathrm{RMSE}}_{k}$)	92.157	103.406	108.703	110.129	108.684	94.898	108.542	77.915	77.491	94.898
	iqr (${\mathrm{RMSE}}_{k}$)	102.432	114.007	119.186	116.515	119.440	153.708	117.857	121.289	120.480	153.708

## Appendix B. Correlations between RMSE an Sequence Parameters

**Table A6:** Correlation between RMSE and entropy of input sequence.

Len	FFNN${}_{\mathrm{lin}}$	FFNN${}_{\tanh}$	LSTM	GRU	BILSTM	LIN	SPLINE	MAKIMA	PCHIP	mSVD
10	0.741	0.878	0.890	0.849	0.890	0.614	0.261	0.552	0.520	0.614
20	0.760	0.827	0.852	0.842	0.790	0.608	0.466	0.550	0.533	0.608
50	0.744	0.851	0.740	0.678	0.670	0.660	0.503	0.603	0.608	0.660
100	0.639	0.719	0.724	0.649	0.662	0.742	0.576	0.661	0.679	0.742
200	0.658	0.691	0.667	0.665	0.664	0.777	0.626	0.756	0.812	0.777
10	0.092	0.021	0.017	0.033	0.017	0.195	0.617	0.256	0.290	0.195
20	0.080	0.042	0.031	0.036	0.061	0.200	0.352	0.258	0.276	0.200
50	0.090	0.032	0.093	0.139	0.146	0.153	0.309	0.205	0.200	0.153
100	0.172	0.108	0.104	0.163	0.152	0.091	0.231	0.153	0.138	0.091
200	0.155	0.129	0.148	0.150	0.150	0.069	0.184	0.082	0.050	0.069

**Table A7:** Correlation between RMSE and standard deviation of input sequence.

Len	FFNN${}_{\mathrm{lin}}$	FFNN${}_{\tanh}$	LSTM	GRU	BILSTM	LIN	SPLINE	MAKIMA	PCHIP	mSVD
10	0.775	0.997	0.950	0.928	0.956	0.729	0.419	0.668	0.586	0.729
20	0.823	0.986	0.969	0.943	0.948	0.688	0.505	0.595	0.556	0.688
50	0.736	0.924	0.703	0.661	0.645	0.718	0.479	0.627	0.614	0.718
100	0.673	0.833	0.755	0.708	0.698	0.747	0.611	0.718	0.707	0.747
200	0.696	0.719	0.649	0.667	0.641	0.648	0.570	0.659	0.694	0.648
10	0.070	0.000	0.004	0.007	0.003	0.100	0.408	0.147	0.222	0.100
20	0.044	0.000	0.001	0.005	0.004	0.131	0.307	0.213	0.252	0.131
50	0.095	0.008	0.119	0.153	0.167	0.108	0.336	0.183	0.195	0.108
100	0.143	0.040	0.083	0.115	0.123	0.088	0.198	0.108	0.116	0.088
200	0.125	0.107	0.163	0.148	0.170	0.164	0.237	0.155	0.126	0.164

**Table A8:** Correlation between RMSE and velocity of input sequence.

Len	FFNN${}_{\mathrm{lin}}$	FFNN${}_{\tanh}$	LSTM	GRU	BILSTM	LIN	SPLINE	MAKIMA	PCHIP	mSVD
10	0.768	0.983	0.943	0.915	0.950	0.701	0.419	0.640	0.564	0.701
20	0.812	0.962	0.950	0.927	0.916	0.669	0.486	0.576	0.540	0.669
50	0.749	0.921	0.724	0.672	0.657	0.716	0.478	0.624	0.619	0.716
100	0.681	0.825	0.772	0.715	0.709	0.771	0.615	0.728	0.723	0.771
200	0.712	0.742	0.679	0.694	0.673	0.710	0.609	0.714	0.755	0.710
10	0.074	0.000	0.005	0.011	0.004	0.121	0.409	0.172	0.243	0.121
20	0.050	0.002	0.004	0.008	0.010	0.146	0.328	0.231	0.269	0.146
50	0.087	0.009	0.104	0.143	0.157	0.110	0.338	0.186	0.190	0.110
100	0.136	0.043	0.072	0.111	0.115	0.072	0.194	0.101	0.104	0.072
200	0.112	0.091	0.138	0.126	0.143	0.114	0.199	0.111	0.083	0.114

**Table A9:** Correlation between RMSE and acceleration of input sequence.

Len	FFNN${}_{\mathrm{lin}}$	FFNN${}_{\tanh}$	LSTM	GRU	BILSTM	LIN	SPLINE	MAKIMA	PCHIP	mSVD
10	0.901	0.867	0.917	0.928	0.922	0.879	0.775	0.853	0.806	0.879
20	0.923	0.853	0.916	0.932	0.894	0.870	0.754	0.806	0.779	0.870
50	0.896	0.952	0.870	0.858	0.846	0.909	0.740	0.845	0.844	0.909
100	0.886	0.960	0.928	0.916	0.907	0.914	0.858	0.926	0.916	0.914
200	0.918	0.929	0.884	0.901	0.879	0.699	0.830	0.789	0.745	0.699
10	0.014	0.025	0.010	0.008	0.009	0.021	0.070	0.031	0.053	0.021
20	0.009	0.031	0.010	0.007	0.016	0.024	0.083	0.053	0.068	0.024
50	0.016	0.003	0.024	0.029	0.034	0.012	0.093	0.034	0.034	0.012
100	0.019	0.002	0.008	0.010	0.012	0.011	0.029	0.008	0.010	0.011
200	0.010	0.007	0.019	0.014	0.021	0.122	0.041	0.062	0.089	0.122

**Table A10:** Correlation between RMSE and jerk of input sequence.

Len	FFNN${}_{\mathrm{lin}}$	FFNN${}_{\tanh}$	LSTM	GRU	BILSTM	LIN	SPLINE	MAKIMA	PCHIP	mSVD
10	0.784	0.711	0.752	0.785	0.760	0.823	0.861	0.818	0.765	0.823
20	0.811	0.723	0.778	0.798	0.784	0.810	0.720	0.750	0.720	0.810
50	0.772	0.813	0.736	0.749	0.737	0.833	0.674	0.770	0.766	0.833
100	0.806	0.881	0.826	0.846	0.827	0.797	0.800	0.855	0.830	0.797
200	0.843	0.843	0.791	0.816	0.785	0.502	0.736	0.625	0.546	0.502
10	0.065	0.113	0.084	0.064	0.080	0.044	0.028	0.047	0.076	0.044
20	0.050	0.104	0.068	0.057	0.065	0.051	0.107	0.086	0.106	0.051
50	0.072	0.049	0.095	0.086	0.095	0.040	0.142	0.073	0.076	0.040
100	0.053	0.020	0.043	0.034	0.042	0.057	0.056	0.030	0.041	0.057
200	0.035	0.035	0.061	0.048	0.064	0.311	0.095	0.185	0.262	0.311

**Table A11:** Correlation between RMSE and monotonicity of input sequence.

Len	FFNN${}_{\mathrm{lin}}$	FFNN${}_{\tanh}$	LSTM	GRU	BILSTM	LIN	SPLINE	MAKIMA	PCHIP	mSVD
10	0.918	0.533	0.722	0.781	0.709	0.952	0.866	0.971	0.993	0.952
20	0.898	0.529	0.694	0.759	0.703	0.971	0.999	0.993	0.996	0.971
50	0.883	0.774	0.857	0.914	0.918	0.937	0.974	0.965	0.953	0.937
100	0.908	0.873	0.853	0.908	0.904	0.817	0.951	0.897	0.890	0.817
200	0.892	0.858	0.866	0.871	0.862	0.441	0.842	0.612	0.476	0.441
10	0.010	0.276	0.106	0.067	0.115	0.003	0.026	0.001	0.000	0.003
20	0.015	0.281	0.126	0.080	0.119	0.001	0.000	0.000	0.000	0.001
50	0.020	0.071	0.029	0.011	0.010	0.006	0.001	0.002	0.003	0.006
100	0.012	0.023	0.031	0.012	0.013	0.047	0.003	0.015	0.018	0.047
200	0.017	0.029	0.026	0.024	0.027	0.381	0.036	0.196	0.340	0.381

**Table A12:** Correlation between RMSE and complexity of input sequence.

Len	FFNN${}_{\mathrm{lin}}$	FFNN${}_{\tanh}$	LSTM	GRU	BILSTM	LIN	SPLINE	MAKIMA	PCHIP	mSVD
10	−0.795	−0.937	−0.913	−0.906	−0.922	−0.781	−0.532	−0.729	−0.645	−0.781
20	−0.837	−0.931	−0.936	−0.920	−0.919	−0.733	−0.568	−0.644	−0.599	−0.733
50	−0.763	−0.914	−0.730	−0.703	−0.687	−0.770	−0.544	−0.685	−0.670	−0.770
100	−0.744	−0.878	−0.802	−0.775	−0.758	−0.787	−0.682	−0.780	−0.759	−0.787
200	−0.754	−0.769	−0.692	−0.714	−0.685	−0.637	−0.618	−0.673	−0.675	−0.637
10	0.059	0.006	0.011	0.013	0.009	0.067	0.278	0.100	0.167	0.067
20	0.038	0.007	0.006	0.009	0.010	0.097	0.239	0.167	0.209	0.097
50	0.078	0.011	0.099	0.119	0.131	0.074	0.265	0.134	0.145	0.074
100	0.090	0.021	0.055	0.070	0.081	0.063	0.135	0.067	0.080	0.063
200	0.083	0.074	0.128	0.111	0.133	0.174	0.191	0.143	0.141	0.174
